# Randomised, crossover clinical trial, in healthy volunteers, to compare the systemic availability of two topical intranasal budesonide formulations

**DOI:** 10.1186/1745-6215-9-34

**Published:** 2008-06-09

**Authors:** Jaime Algorta, Maria Angeles Pena, Silvia Francisco, Zurine Abajo, Emilio Sanz

**Affiliations:** 1 Clinical Trials Unit, Fundacion LEIA-Txagorritxu Hospital, Vitoria-Gasteiz, Spain; 2 University of Basque Country, Spain; 3 European Academy of Allergy and Clinical Immunology, EAACI 2007 Goteborg, Sweden

## Abstract

**Background:**

Budesonide has a long history as intranasal drug, with many marketed products. Efforts should be made to demonstrate the therapeutic equivalence and safety comparability between them. Given that systemic availability significantly varies from formulations, the clinical comparability of diverse products comes to be of clinical interest and a regulatory requirement. The aim of the present study was to compare the systemic availability, pharmacodynamic effect, and safety of two intranasal budesonide formulations for the treatment of rhinitis.

**Methods:**

Eighteen healthy volunteers participated in this randomised, controlled, crossover, clinical trial. On two separated days, subjects received a single dose of 512 μg budesonide (4 puffs *per *nostril) from each of the assayed devices (Budesonida nasal 64^®^, Aldo-Union, Spain and Rhinocort 64^®^, AstraZeneca, Spain). Budesonide availability was determined by the measurement of budesonide plasma concentration. The pharmacodynamic effect on the hypothalamic-adrenal axis was evaluated as both plasma and urine cortisol levels. Adverse events were tabulated and described. Budesonide availability between formulations was compared by the calculation of 90%CI intervals of the ratios of the main pharmacokinetic parameters describing budesonide bioavailability. Plasma cortisol concentration-time curves were compared by means of a GLM for Repeated Measures. Urine cortisol excretion between formulations was compared through the Wilcoxon's test.

**Results:**

All the enroled volunteers successfully completed the study. Pharmacokinetic parameters were comparable in terms of AUC_t _(2.6 ± 1.5 vs 2.2 ± 0.7), AUC_i _(2.9 ± 1.5 vs 2.4 ± 0.7), t_max _(0.4 ± 0.1 vs 0.4 ± 0.2), C_max_/AUC_i _(0.3 ± 0.1 vs 0.3 ± 0.0), and MRT (5.0 ± 1.4 vs 4.5 ± 0.6), but not in the case of C_max _(0.9 ± 0.3 vs 0.7 ± 0.2) and t_1/2 _(3.7 ± 1.8 vs 2.9 ± 0.4). The pharmacodynamic effects, measured as the effect over plasma and urine cortisol, were also comparables between both formulations. No severe adverse events were reported and tolerance was comparable between formulations.

**Conclusion:**

The systemic availability of intranasal budesonide was comparable for both formulations in terms of most pharmacokinetic parameters. The pharmacodynamic effect on hypothalamic-pituitary-adrenal axis was also similar. Side effects were scarce and equivalent between the two products. This methodology to compare different budesonide-containing devices is reliable and easy to perform, and should be recommended for similar products intented to be marketed or already on the market.

**Trial registration:**

No Eudra CT: 2005-003727-39

## Background

Topic intranasal corticosteroids are first-line therapy for allergic and vasomotor rhinitis [1] and among them budesonide is the most prescribed drug because of its demonstrated efficacy and an unequivocal safety profile [2]. These findings are due to its short half-life, tissue retention, topical activity and rapid first-pass hepatic metabolism. Although budesonide has a long history of use as intranasal drug, with many reports confirming its safety [3,4], and since there are many marketed products containing budesonide, efforts should be made for the benefit of patients to demonstrate the therapeutic equivalence and safety comparability between them.

In fact, the most frequent side effects associated with the use of topical budesonide are mainly local effects (such as epistaxis, nasal itching and nasal dryness). However, the most severe adverse effects (adrenal suppression, loss of bone mass and other metabolic and immunologic effects) [5,6] are related to the budesonide into the systemic bloodstream, and is therefore considered to be an undesirable effect in the case of topically acting treatments. Of particular interest is the dose-dependent hypothalamic-pituitary-adrenal axis suppression that occurs with systemic administration of corticosteroids which could, which is much less apparent with the intranasal administration of budesonide.

In the case of intranasal corticosteroids, a high systemic availability could be expected due to the abundant vascularity of the nasal mucosa, and also due to direct absorption from the nasal mucosa which does not provide hepatic first-pass inactivation. In addition, although modern devices deliver most of the administered dose to the nasal mucosa, a percentage of the dose (up to a 50%) [7] is transported by the mechanism of mucociliar clearance to the gastrointestinal tract, where it is ingested and absorbed into the systemic bloodstream.

The systemic bioavailability of nasal corticosteroids is greatly influenced both by the vehicle and by the device characteristics (determining the particle size and the percentage of deposition) [8,9], and therefore, a wide difference could be expected from different manufacturers. Although pressurized aerosols were the first topical formulation to deliver intranasal corticosteroids, over recent years powder formulations and mainly, aqueous pump sprays, are become the usual devices. Aqueous solutions provide an adequate drug deposition in the nasal mucosa and are not accompanied by propellant-associated problems [10].

Some authors [10] have previously demonstrated that systemic availability significantly differs from distinc formulations. Consequently, clinical demonstration of the systemic availability of diverse pharmaceutical formulations and devices becomes of clinical interest and a regulatory requirement. Furthermore, international guidelines [11-13] have established that in the case of local products for the treatment of allergic rhinitis, the systemic absorption and metabolism must be determined. However, systemic availability assessment for locally acting nasal drugs is technically complicated because the administered dose is usually very low and the drug is deposited topically, at the site of action, and its activity does not occur after systemic absortion.

The aim of the present study was to compare the systemic availability, pharmacodynamic effect, and tolerability of two intranasal budesonide formulations for the treatment of allergic or vasomotor rhinitis.

## Methods

### Design and ethics

The present clinical trial is a pharmacokinetic study to evaluate systemic exposure for nasally-acting budesonide-containing products, also including the pharmacodynamic assessment for systemic absorption. The study design was a single-centre, randomised, double-blind, active-controlled, two-way crossover, human pharmacology clinical trial in healthy volunteers.

The trial was designed taking into consideration specific international guidelines [11-15] and was conducted in accordance with the Declaration of Helsinki and the Principles of Good Clinical Practice. The trial was reviewed and approved by the local IEC/IRB (CEIC at Txagorritxu Hospital) and subsequently authorised by the Spanish Medicines Agency. Informed consent was obtained from all the participants before the enrolment in the trial. The trial was registered in the EudraCT database (EudraCT number: 2005-003727-39).

### Subjects

Screening was performed during the 4 weeks preceding the first administration of treatment. Eighteen healthy adult volunteers (10 male and 8 female; mean age 24 years, range 19–39 years) participated in this study at the Clinical Trials Unit (Txagorritxu Hospital, Vitoria, Spain). All of them were Caucasian and had normal body weight and height. Prior to the study, medical history including presence of any allergy or significant disease (cardiac, hepatic, renal pulmonary, neurological, gastrointestinal or haematological), physical examination, electrocardiography, and routine laboratory tests (haematology, clinical chemistry blood test and urinary analysis) were registered. All subjects were negative for hepatitis B, hepatitis C and HIV serology, drug abuse urinary test and pregnancy test (the latter in the case of females). Subjects were not eligible if clinically or analytically relevant results were identified. They were required to abstain from taking any drug, smoking or consuming exciting beverages (including coffee and tea) for two weeks prior to and during the study period. No subject did withdraw from the study and all the enrolled subjects completed the planned schedule.

### Study products

Two different formulations of 64 μg/dose of Budesonide suspension for nasal nebulisation were assayed (Formulation A: Budesonida nasal 64^®^, Aldo-Union, Spain and Formulation B: Rhinocort 64^®^, AstraZeneca, Spain). Following a randomised sequence, subjects received from both devices a single dose of 512 μg budesonide (4 actuations *per *nostril) on two different experimental days, separated by a washout period of at least one week. This supratherapeutic dosing regimen was dispensed to produce a measurable budesonide plasma concentration for estimation of bioavailability and calculation pharmacokinetic of parameters.

Subjects were allocated in experimental groups of 9 individuals. Each investigational day, study drug administration begun at 08:00 with the first volunteer and was sequentially given at 4-minutes intervals under the investigator's direct surveillance. The total dosage was given to each nostril alternatively as eight actuations at intervals of 20 seconds (total time of drug administration, 2 min and 40 s). Time zero for each dose was defined as the time when the inhaler was first actuated.

All inhalers were primed prior to use in a separate room. Before drug dispensation, the subject gently blew his or her nose and afterwards, the subjects rinsed their mouth with water to avoid pharyngeal absorption. Then they remained in relative rest in a semi-recumbent position with the head of the bed elevated at a 45-degree angle for 4 hours.

As part of the screening and before the participation into the study, the subjects were instructed with a placebo on the proper use of the devices and the correct technique of inhalation. Subjects self-administered the treatments, but to assure the compliance and the inhalation technique, the investigator directly supervised this procedure.

### Study development

Budesonide availability was determined by the measurement of budesonide plasma concentration after drug administration. Pharmacodynamic effect on the hypothalamic-adrenal axis was evaluated both through the evaluation of the plasma cortisol and through the comparison of urine cortisol excretion.

Subjects were admitted to the Clinical Trials Unit the evening before each experimental day, when another drug test and pregnancy test (in females) were performed. The next morning, a venous cannula was inserted into a forearm vein and maintained during the session. Blood samples were obtained at times basal (prior to drug administration), +10 min, +20 min, +30 min, +45 min, +1 h, +2 h, +4 h, +6 h, +8 h, +10 h, and +12 h for budesonide measurement. In addition, plasma cortisol concentration was also determined at times basal (clock time extending from 08:00 to 08:32), +2 h, +4 h, +6 h, and +12 h.

For each blood sampling, the first 1.5 ml from the cannula were discarded, then a volume of 8 ml was taken and afterwards 1.5 ml of physiological saline serum were infused to keep the line permeable until next extraction. Urine samples were collected in individual flasks for 12 h after drug administration, the diuresis was also noted and aliquots for urinary cortisol determination were obtained. Both plasma and urine samples were stored frozen at -80°C until analysis. Subjects were required to fast for 10 h before and 4 h after dosing.

### Tolerability assessment

Subjects remained under direct surveillance by the medical staff and safety was monitored throughout the study. All potential adverse events were immediately recorded on the case report form and subsequently evaluated. Vital signs (blood pressure and heart rate) were also monitored throughout the experimental session. In addition, analytical tolerance (haematology, clinical chemistry, and urianalysis) was assessed at screening and at the end of the trial.

### Laboratory measurements

Plasma budesonide was quantified under Good Laboratory Practice by Liquid Chromatography with MS/MS detection, with a limit of quantification (LoQ) of 0.05 ng/ml (ACC GmbH, Germany). Plasma cortisol was quantified by ECLIA quimioluminescent immunoassay with a LoQ of 0.018 μg/dL (E-170 Analyser, Roche, Spain) and urinary cortisol by means of FPIA fluorescent immunoassay with a LoQ of 0.77 μg/dL (TDX/FLX Analyser, Abbot, Spain) at Hospital Txagorritxu, Spain.

### Statistical methods

Considering the intra-individual variability of budesonide plasma levels, for detecting differences of 20% between both formulations with a power of 80% and a significance level of 0.05, and adjusting for a 10% of potential drop-outs, a sample size of 18 subjects was required.

Pharmacokinetic parameters describing budesonide bioavailability in extent and rate were estimated by a non-compartmental model and described as mean ± SD. The maximum concentration (C_max_) and time to reach the maximum concentration (t_max_) were obtained directly from the experimental data. The areas under the curve (AUC_t_, from 0 to the last concentration) were calculated using the linear trapezoidal method and extrapolate to infinity (AUC_i_) using the following formula: AUC_i _= AUC_t_+C_t_/λ_z_. The parameter C_max_/AUC_i _was directly calculated from the data. Elimination half-life (t_1/2_) was calculated by linear regression using the slope of the terminal segment of the curve of log-concentration versus time. Mean residence time (MRT) extrapolated to infinity was calculated using the formula MRT = Area under the moment curve/AUC.

Budesonide availability between formulations was compared by means of a bioequivalence approach. First a log-transformation of the parameters (with the exception of t_max_) was performed, followed by a calculation of 90% Confidence Interval for the ratio of the geometric means for the parameters under consideration, after the administration of each formulation. Considering the nature of the trial and the sample size, a wider interval of 0.75–1.33 was *a priori *considered appropriate. The parameter t_max _was not included in this model because it was considered that the drug absorption rate is better reflected [16] by the parameter C_max_/AUC_i_, with the advantage that its analysis is possible under this approach. Then, in the case of t_max_, the 90% Confidence Interval for the difference of the means was calculated by the non-parametric Hauschke's method.

Pharmacodynamic effect was evaluated through the measurements of plasma cortisol (at times 0, 2, 4, 8 and 12 h) and urinary cortisol excretion during the 12 h after drug administration. The time-concentration curves of plasma cortisol were first described and subsequently compared by means of a General Linear Model for Repeated Measures procedure (SPSS^®^), with Time as within-subjects factor (5 levels) and Formulation as between-subjects factor (2 levels). The parameter AUC was also calculated for plasma cortisol and subsequently compared by means of the non-parametric Wilcoxon's test for paired data. Urine cortisol excretion between formulations was compared by means of the Wilcoxon's test. Adverse events were tabulated and described.

## Results

All the eighteen normal volunteers who entered in the study successfully completed the trial and none discontinued.

### Systemic availability of Budesonide

**Figure 1:**
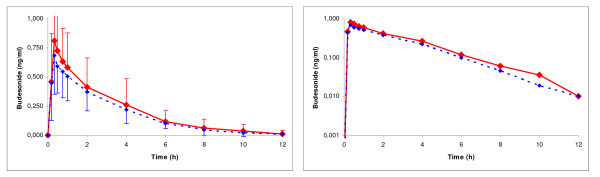
Mean ± SD plasma budesonide concentrations after the administration of Formulation A () or Formulation B (). Left, Concentration/Time curve. Right, Log-concentration/Time curve.

Concentration/time and log-concentration/time curves of systemic budesonide availability are shown in Figure 1, exhibiting the evident similarity of the plasma level profiles. Table 1 summarises the main pharmacokinetic parameters describing the systemic absortion in extent and rate.

**Table 1:** Pharmacokinetic parameters (expressed as mean ± SD) after the intranasal administration of 512 μg budesonide

	**C**_**max **_(ng/ml)	**AUC**_**t **_(h*ng/ml)	**AUC**_**i **_(h*ng/ml)	**t**_**max **_(h)	**C**_**max**_**/AUC**_**i **_(ng/ml)/(h*ng/ml)	**t**_**1/2 **_(h)	**MRT **(h)
**Formulation A**	0.90 ± 0.33	2.61 ± 1.50	2.91 ± 1.54	0.43 ± 0.18	0.33 ± 0.10	3.79 ± 1.83	5.01 ± 1.40
**Formulation B**	0.77 ± 0.24	2.24 ± 0.78	2.46 ± 0.77	0.43 ± 0.25	0.32 ± 0.08	2.98 ± 0.42	4.57 ± 0.69

Comparison between formulations was performed through a bioequivalence approach as can be seen in Table 2. Most of the parameters can be considered as equivalent since they fall within the acceptance range *a priori *established of the 0.75–1.33 of 90% Confidence Intervals, with the exception of C_max _and t_1/2 _that exhibited a slight increase in the case of formulation A.

**Table 2:** Bioequivalence evaluation (expressed as 90%CI of the Ln-transformed ratio, except for tmax, which is expressed as 90%CI of the untransformed differences)

	**Ratio**	**90%Confidence Interval**
**Ln(C_**max**_)**	1.13	0.95 – 1.35
**Ln(AUC_**t**_)**	1.10	0.92 – 1.32
**Ln(AUC_**i**_)**	1.12	0.95 – 1.32
**Ln(C_**max**_/AUC_**i**_)**	1.01	0.89 – 1.14
**Ln(t_**1/2**_)**	1.18	1.02 – 1.37
**Ln(MRT)**	1.07	0.97 – 1.20
**t_**max**_**	1.05	0.86 – 1.29

### Pharmacodynamic effect

**Figure 2:**
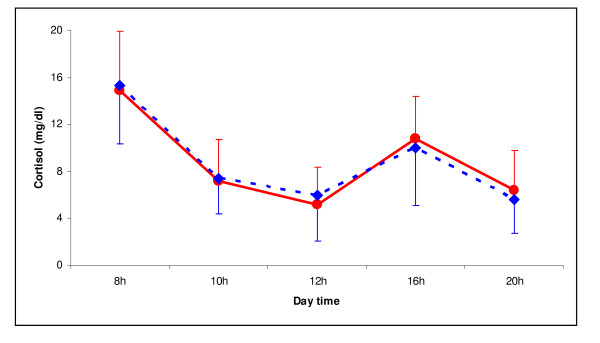
Mean ± SD plasma cortisol concentrations after the administration of Formulation A () or Formulation B ().

The systemic effect of budesonide was evaluated through the evaluation of its effect over plasma and urine cortisol. Figure 2 shows the plasma cortisol concentration/time curve. No significant differences are shown when the curves are analyzed, neither by means of a repeated measures model nor through AUC comparison (Formulation A: 100.9 ± 20.0 mg/dl; Formulation B: 99.6 ± 27.6 mg/dl). Table 3 shows the diuresis and cortisol excretion during the 12 h after drug administration. No significant differences are seen between both formulations.

**Table 3:** Cortisol excretion (expressed as mean ± SD)

	**Diuresis **(ml/12 h)	**Cortisol concentration **(μg/dl)	**Cortisol Excretion **(μg/12)
**Formulation A**	636 ± 280	20.6 ± 9.2	120 ± 51
**Formulation B**	774 ± 297	18.9 ± 5.9	134 ± 37

### Tolerability

No serious adverse events occurred during the trial. A total of 11 side-effects were considered and occurred in 7 of the 18 volunteers. In four of the subjects, events were recorded with either formulation A and B. The most frequently evidenced events were oropharyngeal dryness (2 cases with formulation A and 2 cases with formulation B) and headache (1 case with formulation A and 2 cases with B). Other occurrences were episodes of pharyngitis (formulation A), pruritus (2 cases with formulation B) and lymphopaenia (formulation B).

## Discussion

Topical administration of corticosteroids is preferred over systemic administration to localize their effect, reduce the total dose required and to minimize side effects. The present study evaluates the systemic availability, pharmacodynamic effect and tolerance of two intranasal formulations of budesonide. Systemic absorption can either occur from the nasal mucosa, thereby reflecting the amount of drug exerting the therapeutic effec, or from the gastrointestinal tract. Although intranasal budesonide is a locally applied locally acting drug, investigation of systemic availability is recommendable since the most important side effects are associated with the undesirable pass of budesonide from nasal mucosa (or gastric mucosa) to the general circulation, and its effect mainly on the hypothalamic-pituitary-adrenal axis suppression. This is of particular relevance in the case of budesonide, which is better absorbed through the nasal mucosa than other corticosteroids (e.g., fluticasone or mometasone).

Many factors can influence the variability that would be seen within dosing of budesonide in clinical practice, comprinsing both inter-individual variability and inter-products disparity. The rationale for investigating two distinc products is based in the broad difference in systemic availability which can be expected from diverse manufacturers, due to either product excipients or device characteristics. Since several different products are available on the market and it is a common clinical practice to switch between them, all marketed products should be compared to preserve patient's safety. *In vitro *methods to evaluate nasal sprays or aerosols (e.g., the characterisation of particle size distribution, plume geometry, etc.) have clear advantages compare with *in vivo *(less variability, facility to control), and are indispensable during the product development to demonstrate equivalent performance. Nevertheless, in addition to *in vitro *studies, clinical studies are also essential to detect differences between products, especially due to the difficulties encountered in adequately characterising drug particle size distribution [13].

Clinical studies can be carried out either in healthy volunteers or in allergic rhinitis patients. However, since the effect of inflammation and swelling in the mucosal lining of the nose on systemic availability in patients with rhinitis is unknown and could potentially increase the inter-subjects variability, the study design with the enrolment of healthy volunteers was considered the most appropriate model [13]. Moreover, the systemic availability of budesonide was previously evaluated in healthy subjects by other authors [10].

In this clinical study, the main pharmacokinetic parameters after a single dose of 512 μg budesonide given by two different devices were estimated. Results were consistent with previous communications by others [10], when a similar C_max _of 0.43 ng/ml (0.99 nmol/l) was evidenced after a single dose of 400 μg. Also, the rapid plasma clearance is compatible with the expected rapid first-pass hepatic metabolism.

In addition, the two products were compared in terms of systemic bioavailability, pharmacodynamic effect and tolerance. Although pharmacokinetic parameters are extensively described and the comparison is based on the calculation of ratios and confidence intervals, this research can not be considered a true bioequivalence as for orally administered drugs, but a way to investigate the systemic availability (and therefore, the likelihood of producing systemic adverse events) of a locally-acting drug. In this case, both products are revealed to be having a very similar systemic exposure, in terms of extent and rate. Only in two (C_max _and t_1/2_) out of seven pharmacokinetic parameters analysed is the upper 90% confidence interval slightly exceeded, even though this difference was not reflected in the pharmacodynamic effect or in the tolerance, where no differences were revealed. Nevertheless, in the case of highly variable products, wider acceptance intervals are accepted, even 0.70–1.42, wider than the assumed in our study [17].

Plasma levels were variable, as would be expected for intranasally administered drugs. The high variability found for those products can be elevated due to individual characteristics such as variations in nasal anatomy or mucoliciar drainage. Another factor potentially contributing to the elevated variability is the possible alteration of the drug deposition pattern because of the experimentally higher volume admistered in this study, and the greater loss of drug into the nasypharynx or externally from the nasal cavity. What should also be considered is that the relative scarce sample size also contributes to the presence of a wider confidence interval. However, the most robust parameters defining bioavailability (principally AUC, but also MRT) are absolutely comparable between both formulations and devices.

In accordance with international guidelines, in the case of products for local use (such as nasal administration), pharmacodynamic clinical studies are recommended in addition to the clinical studies needed to investigate equi-effectiveness. Adrenal suppression is the main pharmacodynamic effect of corticoids and has been used as a marker for the systemic bioactivity of topic corticosteroids [9]. In the present study, adrenal suppression during drug exposure was assessed by both plasma cortisol measurement and 12-hour fractionated urine cortisol excretion. Both products demonstrated analogous effect of plasma or urine cortisol, but noteworthy is the fact that adrenal suppression was minor, in spite of the high dose of budesonide administered (512 μg), 4-fold higher than that usually recommended for the clinical use (64 μg/nostril), and it is an important safety finding. In addition, both formulations were clinically and analytically well tolerated and only minor discomfort episodes were reported, in similar number and severity with both formulations.

## Conclusion

A complete description of pharmacokinetics parameters after a single dose of intranasal budesonide is given and two different intranasal devices are compared in terms of systemic budesonide availability, pharmacodynamic effect and tolerance, concluding equivalence between them. This methodology for comparing different budesonide-containing devices is reliable and easy to perform, and should, together with clinical efficacy studies, be recommended for all similar products intended to be marketed or already on the market.

## Competing interests

The trial was sponsored by Laboratorio Aldo-Union, Spain. The authors declare no competing interests.

## Authors' contributions

JA wrote the manuscript participated in the design and coordinated the study, MAP, SF, and ES participated in the design and development of the study, JA and MAP performed the statistical analysis. All authors revised the manuscript and approved the final version of the manuscript, ZA contributed to the design of the study and was responsible for overseeing the application of the protocol by monitoring the trial and to ensuring compliance with the Good Clinical Practice guidelines.
